# Magnetoreception in birds: II. Behavioural experiments concerning the cryptochrome cycle

**DOI:** 10.1242/jeb.110981

**Published:** 2014-12-01

**Authors:** Roswitha Wiltschko, Dennis Gehring, Susanne Denzau, Christine Nießner, Wolfgang Wiltschko

**Affiliations:** Goethe-Universität Frankfurt, FB Biowissenschaften, Max-von-Laue-Straße 13, D-60438 Frankfurt am Main, Germany

**Keywords:** Cryptochrome 1a, Flavin cycle, Photoreduction, Activated Cry1a, Radical pair mechanism, Migratory orientation

## Abstract

Behavioural tests of the magnetic compass of birds and corresponding immunohistological studies on the activation of retinal cryptochrome 1a, the putative receptor molecule, showed oriented behaviour and activated Cry1a under 373 nm UV, 424 nm blue, 502 nm turquoise and 565 nm green light, although the last wavelength does not allow the first step of photoreduction of cryptochrome to the semiquinone form. The tested birds had been kept under ‘white’ light before, hence we suggested that there was a supply of semiquinone present at the beginning of the exposure to green light that could be further reduced and then re-oxidized. To test the hypothesis in behavioural experiments, we tested robins, *Erithacus rubecula*, under various wavelengths (1) after 1 h pre-exposure to total darkness and (2) after 1 h pre-exposure to the same light as used in the test. The birds were oriented under blue and turquoise light, where the full cryptochrome cycle can run, but not under green light. This finding is in agreement with the hypothesis. Orientation under green light appears to be a transient phenomenon until the supply of semiquinone is depleted.

## INTRODUCTION

The Radical Pair Model (Ritz et al., 2000) proposed that the avian magnetic compass is based on radical pair processes in the eye. Cryptochrome, a blue light receptor with flavin as the chromophore (for review, see Chaves et al., 2011) was suggested as the receptor molecule that generated the radical pairs. A type of cryptochrome, Cry1a, was found in the eyes of birds, where it is located at the discs of the outer segment of the UV/violet cones of European robins, *Erithacus rubecula* (Turdidae), and domestic chickens, *Gallus gallus* (Phasianidae) (Nießner et al., 2011). Behavioural experiments testing migratory birds in cages are in accordance with a role of Cry1a in magnetoreception: Australian silvereyes, *Zosterops l. lateralis* (Zosteropidae), European robins and garden warblers, *Sylvia borin* (Sylviidae), were found to be oriented in their seasonally appropriate migratory direction under 373 nm UV, 424 nm blue, 502 nm turquoise and 565 green light; under longer wavelengths, they were disoriented (Wiltschko et al., 1993; Wiltschko and Wiltschko, 1995; Wiltschko and Wiltschko, 1999; Rappl et al., 2000; Muheim et al., 2002; Wiltschko et al., 2014). This is in agreement with the absorption spectrum of cryptochrome: the oxidized form absorbs UV and blue light up to about 500 nm, the photoreduced semiquinone form additionally absorbs green light up to about 570 nm (Müller and Ahmad, 2011).

An immunohistochemical study (Nießner et al., 2013) showed that the specific antiserum used only labelled activated Cry1a. With this tool, it could be shown that labelled Cry1a in chicken was found after exposure to the same light that had been used in the behavioural tests with migratory birds. In particular, activation under green light, which is matched by oriented behaviour by the inclination compass in behavioural tests (see Wiltschko et al., 2001), seemed odd, because this wavelength does not allow the first step of photoreduction of cryptochrome. A detailed discussion of the flavin cycle of cryptochrome involved in magnetoreception in birds is given in our previous paper (Nießner et al., 2013); the findings seemed to indicate that the re-oxidation of the fully reduced form and the radical pair generated (see Müller and Ahmad, 2011) is essential for magnetoreception. As the first step of photoreduction from the fully oxidized form to the semiquinone cannot take place under green light, we suggested that the time before exposure to green light was crucial: when the birds had been kept under ‘white’ light, a certain supply of the semiquinone was left; this could be photoreduced to the fully reduced form also under green light, and then re-oxidized (a reaction that is independent of light), generating the magneto-sensitive radical pairs.

To test this assumption, we performed another immunohistochemical study where we pre-exposed chickens to darkness before exposing them to the narrow-band test lights, and also exposed them to the test lights for twice as long as before. The results, reported in the accompanying paper (Nießner et al., 2014) support our hypothesis: under 373 nm UV, 424 nm blue and 502 nm turquoise light, that is, under wavelengths that can photoreduce oxidized cryptochrome, we found activated Cry1a; under 565 nm green light, in contrast, no Cry1a was labelled. Obviously, in the dark and during the longer exposure to green light, the supply of semiquinone had been depleted and the fully reduced Cry1a re-oxidized so that no protein was left in the activated form.

Here, we report the results of corresponding behavioural experiments with European robins, *E. rubecula* (Linnaeus 1758), that (1) were pre-exposed for 1 h to total darkness before being tested under the respective lights, and (2) were tested twice, first for 1 h after coming from the white light in the birds' room, then immediately afterwards again for 1 h in the same light as during the first hour. The test lights were the same narrow-band lights produced by light-emitting diodes (LEDs) used in previous behavioural (see e.g. Wiltschko et al., 2010) and immunohistochemical studies (Nießner et al., 2013; Nießner et al., 2014).

**Table 1.**
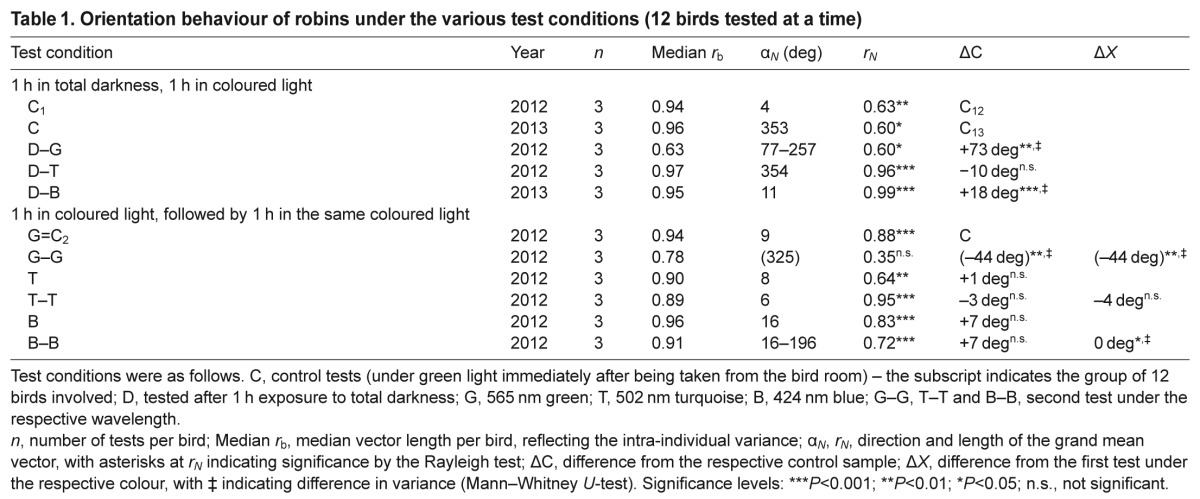
Orientation behaviour of robins under the various test conditions (12 birds tested at a time)

## RESULTS

The numerical results of the various test conditions and the differences from the control samples are listed in Table 1, with significance levels indicated; for the performance of the individual birds, see supplementary material Tables S1–S4.

### Pre-exposure to total darkness

**Fig. 1.**
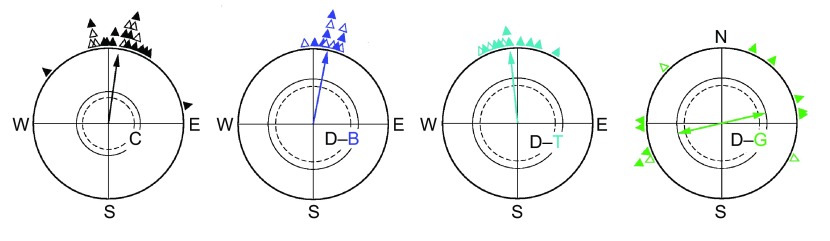
**Orientation behaviour of European robins in light of different wavelengths after being kept in total darkness for 1 h.** C, control under green light, after being in the ‘white’ light of the bird room. D, darkness followed by exposure to the respective light: B, 424 nm blue; T, 502 nm turquoise; G, 565 nm green light. The triangles at the periphery of the circles mark the mean headings of the individual birds tested three times in the respective condition: solid symbols, unimodal mean headings; open symbols, preferred end of an axis. The arrow represents the mean vector or the mean axis, and the two inner circles indicate the 5% (dotted) and the 1% significance border of the Rayleigh test.

The orientation of robins after 1 h pre-exposure to total darkness is shown in Fig. 1. In the control condition, the birds significantly preferred their seasonally appropriate northern migratory direction. They were also oriented in their migratory direction under blue and turquoise light. Under green light, in contrast, the birds were not oriented in their migratory direction, but instead showed a tendency along the east–west axis, with the distribution of the birds' headings significantly different from the control tests (Table 1).

### Testing the birds twice under the same wavelength of light

**Fig. 2.**
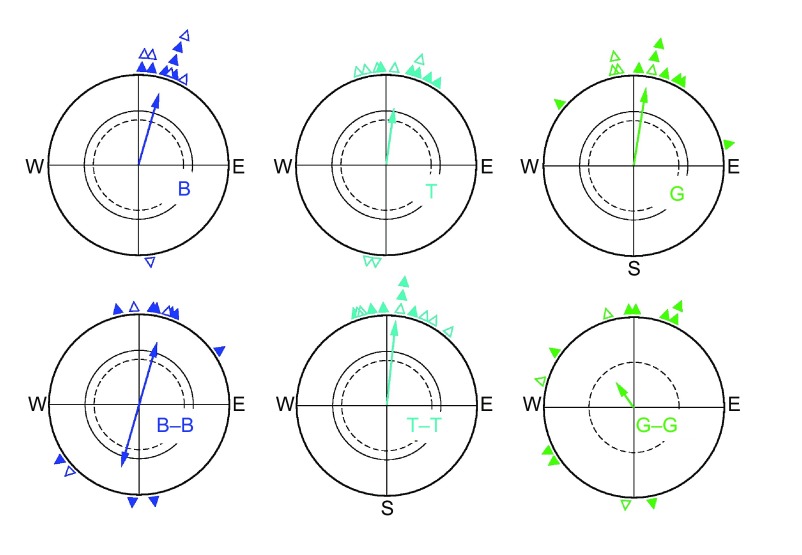
**Orientation behaviour of robins in light of different wavelengths in two consecutive tests of 1 h each, one immediately following the other.** Top row: first test with the light: B, 424 nm blue; T, 502 nm turquoise; G, 565 nm green light. Bottom row: second test in the same light. Symbols are as in Fig. 1.

The orientation of the robins in the two tests under the same light is given in Fig. 2. During the first hour, the robins were significantly oriented in their migratory direction in all light conditions, including green light (Fig. 2, top row). During the second tests immediately following, in the second hour, the birds showed a preference for the migratory axis under blue light and normal migratory orientation under turquoise light; under green light, however, they were disoriented, with the distribution of their mean headings significantly more scattered than during the first hour (Fig. 2, bottom row).

In summary, while the birds always showed orientation in their migratory direction or axis under blue and turquoise light, under green light, in contrast, they were oriented only when they had been in ‘white’ light before. After having stayed for 1 h in total darkness or in green light, the magnetic compass was disrupted, as their disorientation indicates.

## DISCUSSION

Pre-exposure to light conditions other than ‘white’ light affects subsequent orientation behaviour, depending on the wavelength of the test light and the redox cycle of cryptochrome. Our interpretation of these data must necessarily be rather speculative, but here we present the version that seems most likely to us.

While the full redox cycle of cryptochrome can run in UV, blue and turquoise light, the first step from the fully oxidized cryptochrome to the semiquinone cannot take place in green light. Nevertheless, the robins are well oriented if they have been in ‘white’ light before. The disorientation observed under green light after 1 h pre-exposure to total darkness or after having been under the same green light for 1 h beforehand emphasizes the importance of a preceding phase under ‘white’ light where all forms of cryptochrome are present at the same time, in a dynamic equilibrium determined by the light intensity. When the birds are then exposed to narrow band green light, the semiquinone can no longer be formed, but the supply of semiquinone present enables the second part of the cycle to run to the fully reduced form and subsequent re-oxidation.

This appears sufficient for magnetoreception at the beginning of the tests, when the crucial orientation process takes place (see Wiltschko and Wiltschko, 1978; Viehmann, 1982), as reflected by the oriented behaviour of birds. After 1 h, however, any semiquinone available in the beginning appears to have been exhausted – in the dark, it was directly re-oxidized, whereas under green light, it was in part further reduced and then re-oxidized (see Müller and Ahmad, 2011). This means that the processes providing birds with directional information cannot run any longer and, consequently, the robins are disoriented.

The behaviour in green light after pre-exposure to darkness or green light observed here supports the hypothesis that when coming from ‘white’ light there is indeed a certain supply of semiquinone present that can be further photoreduced, allowing orientation as long as there is a sufficient amount left. This is also in accordance with the idea that it is not the radical pair FADH^•^/Trp^•^ generated during the first step of photoreduction – which cannot be formed in green light – but the one formed during re-oxidation of the fully reduced form that is the crucial one for magnetoreception (see Nießner et al., 2013). This is unusual insofar as FADH^•^/Trp^•^ is mostly considered to be the one that mediates the information provided by cryptochrome (e.g. Berndt et al., 2007; Banerjee et al., 2007; Bouly et al., 2007), but possibly the fact that in this case cryptochrome does not signal the presence and amount of light but, rather, the direction of the magnetic field that makes the second radical pair more suitable (see Ritz et al., 2009; Lee et al., 2014). Our findings are in good agreement with immunohistochemical studies on the cryptochrome cycle which clearly show that after 30 min illumination with green light, there is still some activated Cry1a to be found (Nießner et al., 2013), while after 1 h, activated Cry1a is no longer present (Nießner et al., 2014).

Orientation under green light thus appears to be a transient phenomenon. It is almost ironical that we have used this green light as a control condition in all our orientation studies since 2001 with light of various wavelengths (see Wiltschko et al., 2010), in the experiments with radio frequency fields (e.g. Ritz et al., 2004; Ritz et al., 2009) and in those on lateralization (Wiltschko et al., 2002; Gehring et al., 2012). When we began testing birds in different light regimes in the early 1990s, we could only buy two types of LEDs – 565 nm green and 635 nm red. Under green light, the birds were well oriented, while under red light, they were disoriented (Wiltschko et al., 1993). So, we later chose green light for our control, and the relatively short testing times – initially 75 min, later 60 min – ensured that we observed well-oriented behaviour in our test cages.

## MATERIALS AND METHODS

The experiments reported here were performed in spring 2012 and 2013 in accordance with the rules and regulations of animal welfare in Germany.

The test birds were young robins that had been caught during the previous autumn as transmigrants of probably Scandinavian origin in the Botanical Garden of Frankfurt am Main (50°08′N, 8°40′E). During the winter they were kept in a bird room lit by ‘white’ fluorescent light that included wavelengths in the near-UV. By prolonging the photoperiod from 8 h:16 h light:dark to 13 h:11 h around New Year, migratory activity was induced at the beginning of January so that the birds could be tested in spring migration mood in January and February.

All tests took place in wooden huts in the garden of the Zoological Institute where the local geomagnetic field was largely undisturbed. The test lights were produced by LEDs, with a quantal flux of about 8×10^15^ quanta s^−1^ m^−2^; for details, see table 1 in the companion paper (Nießner et al., 2014). The light in the test cages was controlled with a radiometer (Optometer P-9710-1, Gigahertz Optik, Puchheim, Germany) before each test.

For the series with pre-exposure to darkness, the test birds were caught in their housing cages in the evening shortly before the room lights went off, placed in a small closed chamber in a wooden transportation cage, transported to the dark test hut and left there for 1 h before they were placed in the orientation cages illuminated by the LEDs. Testing lasted 1 h. As a control, we used our standard control condition, namely tests of the same birds under 565 nm green light immediately after being brought from the housing cages in the bird room. In the series with two tests following one another, the first test started at the normal time the lights went off in the bird room; after 1 h of testing, the birds were removed from the test cage and placed in a second one illuminated with the same narrow-band light used in the first test. This was done to enforce another, independent orientation process in a new environment. The transfer took place under dim light from the LEDs used to illuminate the cage during testing.

Testing and data analysis followed our standard protocol (e.g. Wiltschko et al., 2014): the birds were tested one at a time in funnel-shaped cages, three times in each condition. The inclined walls of the cages were lined with thermo paper (Mouritsen et al., 2009) where the birds left marks as they moved. To obtain the birds' directional preferences, the thermo paper was removed, divided into 24 sectors, and the marks in each sector were counted by a person who was blind to the test conditions. Recordings with a total of fewer than 35 scratches were excluded from the analysis because of too little activity, and the birds were tested again.

From the distribution of the activity within the cage, the heading of the bird in the respective test was calculated by vector addition. There was a high number of recordings where the bird showed an axial preference; that is, there was a peak of activity in one direction and another one roughly opposite; hence, we applied the procedure described in detail in two previous papers (Wiltschko et al., 2007; Wiltschko et al., 2014). We calculated the unimodal headings and also, by doubling the angles, the axial headings of each recording. For further analysis, we used the heading that produced the longer vector; if this was the axis, we entered the end with more activity into the next step of the analysis. From the three headings of a bird under each condition, we calculated the mean vector of that bird with heading α_b_ and length *r*_b_. For birds that showed axial behaviour, we calculated the axial vector. The mean headings or ends of the mean axes α_b_ of all test birds were then combined into the grand mean vector for each condition, with direction α*_N_* and length *r_N_*, and, by doubling the angles, a grand mean axis, which were tested by the Rayleigh test for significant directional preferences (Batschelet, 1981). This evaluation procedure was applied to all data, including those with only occasional axiality.

The data from different test conditions were compared with the non-parametric Mardia–Watson–Wheeler test to look for differences in distribution, and with the Mann–Whitney test applied to the differences of the birds' mean headings α_b_ from the grand mean to look for differences in variance. We also calculated the median of the vector lengths *r*_b_ per bird, which reflects the intra-individual variance.

Supplementary material Tables S1–S4 give the mean headings and the vector lengths of the individual test birds.

## Supplementary Material

Supplementary Material
