# Supplementary material for: Magnetoreception in birds: II. Behavioural experiments concerning the cryptochrome cycle
Source: J Exp Biol. 2014 Dec 1;217(23):4225–8. doi: 10.1242/jeb.110981 (PMC4254397; doi:10.1242/jeb.110981)
Supplement: Supplementary Material [file supp_217_23_4225__index.html]

Magnetoreception in birds: II. Behavioural experiments concerning the cryptochrome cycle — Supplementary Material 

# Magnetoreception in birds: II. Behavioural experiments concerning the cryptochrome cycle

## JEB110981 Supplementary Material

**Files in this Data Supplement:**

- **Supplementary Material**
