# Supplementary material for: Magnetoreception in birds: II. Behavioural experiments concerning the cryptochrome cycle
Source: J Exp Biol. 2014 Dec 1;217(23):4225–8. doi: 10.1242/jeb.110981 (PMC4254397; doi:10.1242/jeb.110981)
Supplement: Supplementary Material [file supp_217.23.4225_JEB110981.pdf]

Supplementary Material

The orientation behaviour of individual birds

(1) Birds tested for 1 h after 1 h pre-exposure in darkness

Table S1. Birds tested in spring 2012

Bird	Control		D-G		D-T	
	α_b	r_b	α_b	r_b	α_b	r_b
11-1	333°	0.98 [A]	249°	0.56	353°	0.98
11-2	42°	0.77 [A]	272°	0.43	29°	0.99
11-3	198°	0.87 [A]	267°	0.62	340°	0.82 [A]
11-4	360°	0.98	39°	0.88	15°	0.98
11-5	184°	0.94 [A]	244°	0.56	2°	0.99
11-6	360°	0.96 [A]	243°	0.09 [A]	333°	0.22 [A]
11-26	4°	0.95	116°	0.6 4 [A]	9°	0.99
11-8	11°	1.00	23°	0.73	344°	0.83
11-9	346°	0.79	82°	0.55	347°	0.89
11-10	25°	0.50 [A]	72°	0.94	354°	0.99 [A]
11-11	6°	0.93 [A]	314°	0.86 [A]	336°	0.84 [A]
11-12	7°	0.94	84°	1.00	350°	0.96 [A]

Control, tests in 565 nm green light immediately after coming from ‘white’ light; D-G, D-T, tested in 565 nm green and 502 nm turquoise after 1 h pre-exposure in total darkness. - α_b, r_b, direction and length of the birds’ mean vectors based om 3 recordings each, with [A] at the vector lengths indicating the preferred end of an axis.

Table S2. Birds tested in spring 2013

Bird	Control		D-B	
	α_b	r_b	α_b	r_b
12-1	19°	0.98	354°	0.99 [A]
12-2	4°	0.92	9°	0.77 [A]
12-4	15°	1.00 [A]	20°	0.57 [A]
12-5	359°	0.71	18°	0.97 [A]
12-6	350°	0.74 [A]	19°	0.93
12-7	14°	0.81	10°	0.97 [A]
12-8	359°	1.00	10°	0.96
12-9	356°	0.83	7°	0.99
12-25	357°	0.89	1°	0.89
12-26	24°	0.84	10°	0.94
12-27	15°	0.59	17°	0.85
12-29	14°	0.65 [A]	14°	0.94 [A]

D-B, tested in 424 nm blue after 1 h pre-exposure in total darkness. Other abbreviations as in Table S1.

(2) Birds tested twice in the same narrow-band light

Table S3. Bird orientation during the 1st h of the double tests

Bird	G = Control		T		B	
	α_b	r_b	α_b	r_b	α_b	r_b
11-27	351°	0.93 [A]	343°	0.08 [A]	1°	0.60 [A]
11-14	11°	0.98	26°	0.93	7°	0.88
11-15	77°	0.60	357°	0.90	8°	0.89
11-16	348°	0.94 [A]	191°	0.90 [A]	2°	0.98
11-17	30°	0.85	348°	0.48 [A]	19°	0.99 [A]
11-18	12°	0.98	18°	0.95 [A]	22°	1.00
11-19	11°	0.99 [A]	355°	0.76 [A]	16°	1.00
11-20	310°	0.53	5°	0.98 [A]	16°	0.95
11-21	19°	0.98	32°	0.91	19°	0.97
11-22	350°	0.79 [A]	186°	0.74 [A]	20°	0.52 [A]
11-23	3°	0.97	15°	0.65	28°	0.96 [A]
11-24	27°	0.91	19°	0.95	173°	0.87 [A]

G, 565 nm green, T, 502 nm turquoise, B, 424 nm blue. Other abbreviations as in Table S1.

Table S4. Bird orientation during the 2nd h of the double tests

Bird	G-G		T-T		B-B	
	α_b	r_b	α_b	r_b	α_b	r_b
11-27	286°	0.79 [A]	8°	0.92	226°	0.83 [A]
11-14	236°	0.83	358°	0.99	10°	0.82
11-15	243°	0.76	341°	0.90	235°	0.98
11-16	24°	0.75	8°	0.88	56°	0.72
11-17	357°	0.93	29°	0.60 [A]	357°	0.92 [A]
11-18	25°	0.74	345°	0.98 [A]	17°	0.64 [A]
11-19	185°	0.78 [A]	8°	0.73 [A]	19°	0.99
11-20	305°	0.62	23°	0.86 [A]	347°	0.72
11-21	344°	0.37 [A]	351°	0.93	8°	0.97
11-22	169°	0.77	40°	0.78	184°	0.90
11-23	1°	0.92	343°	0.75 [A]	22°	1.00
11-24	19°	0.94	16°	0.96	171°	0.98 [A]

Abbreviations as in Table S3.
